# Lycopene Alleviates Deoxynivalenol-Induced Porcine Intestinal Epithelial Barrier Injury by Inhibiting PGAM5-Mediated Mitophagy-Dependent Ferroptosis

**DOI:** 10.34133/research.1251

**Published:** 2026-04-22

**Authors:** Jing Zheng, Zi-Yan Hu, Ming Lou, Xin Yao, Yue Cheng, Yi-Feng Huang, Ming-Shan Chen, Jia-Xin Wang, Fu-Wei Jiang, Yi Zhang, Zhuo-Yu Liu, Si-Tong Liu, Hong-Li Si, Qi Yu, Xiao-Yi Zhang, Jin-Long Li, Yi Zhao

**Affiliations:** ^1^ College of Veterinary Medicine, Northeast Agricultural University, Harbin 150030, P.R. China.; ^2^ Key Laboratory of the Provincial Education Department of Heilongjiang for Common Animal Disease Prevention and Treatment, Northeast Agricultural University, Harbin 150030, P.R. China.; ^3^ Heilongjiang Key Laboratory for Laboratory Animals and Comparative Medicine, Northeast Agricultural University, Harbin 150030, P.R. China.

## Abstract

Deoxynivalenol (DON), a mycotoxin produced by *Fusarium* species, is a major and unavoidable environmental contaminant that poses serious risks to intestinal health. Lycopene (LYC), a natural carotenoid with potent antioxidant properties, has been reported to exert protective effects against oxidative stress. Phosphoglycerate mutase family member 5 (PGAM5) acts as a key signaling hub to control mitochondrial dynamics and mitophagy. This study aimed to elucidate the potential role of LYC in DON-induced intestinal damage and clarify the contribution of PGAM5. We established intestinal porcine epithelial cell models to explore the effects of DON and LYC on intestinal barrier integrity, mitochondrial function, mitophagy, and ferroptosis through assessments of cell viability, oxidative stress, iron accumulation, and autophagic activity. Mechanistic insights were validated using RNA sequencing, molecular docking, Western blotting, and immunofluorescence analyses. PGAM5 expression was modulated via plasmids and small interfering RNA. Our results demonstrated that DON disrupted barrier integrity, reduced cell motility, and induced cytoskeletal disorganization, accompanied by excessive mitophagy, lipid peroxidation, and ferrous iron accumulation, ultimately leading to ferroptosis. Notably, LYC alleviated DON-induced intestinal damage by inhibiting mitophagy and ferroptosis. Importantly, PGAM5 overexpression abolished the protective effects of LYC, indicating that PGAM5-mediated mitophagy-dependent ferroptosis plays a critical role in DON-induced intestinal damage. These findings suggest that LYC may serve as a potential therapeutic strategy for treating mycotoxin-induced intestinal disorders.

## Introduction

Mycotoxins are secondary metabolites that naturally develop during the stages of crop growth, storage, transportation, and processing, posing serious risks to human health [1,2]. Recent monitoring studies have demonstrated that mycotoxin contamination of global food and feed is highly prevalent [3,4]. It has been reported that the most common mycotoxins in feed components were detected in different Chinese regions from 2018 to 2020, suggesting that deoxynivalenol (DON), as a mycotoxin generated by *Fusarium* species, is among the most frequently found contaminants in food and feed [5,6]. Nationwide surveys from 2021 to 2024 indicated that the occurrence of DON ranged widely from 33.3% to 100% in raw ingredients, with 1.1% of complete feed samples exceeding the national safety limits [7]. DON is highly chemically and thermally stable during food processing, leading to its frequent occurrence [8]. Consequently, DON persists and accumulates in the food chain after it enters the environment, which results in considerable health hazards [9]. Emerging evidence has indicated that the ingestion of feeds contaminated with DON may trigger toxic effects, such as intestinal toxicity and immunotoxicity [10]. Moreover, DON is quickly absorbed following oral intake and quickly spreads into different organs and tissues, resulting in severe organ damage [11,12]. Despite extensive research on DON-induced intestinal toxicity, its molecular mechanisms are still limited and require further study.

Lycopene (LYC), a carotenoid characterized by 11 conjugated double bonds, is abundantly present in red foods including tomatoes, pomegranate, and pink grapefruit [13]. LYC is recognized as a powerful antioxidant because of its strong free radical scavenging activity and superior singlet oxygen quenching capacity [14]. Increasing evidence has shown that LYC is known to have pharmacological properties, including anti-inflammatory, antioxidant, and antiapoptotic effects [15]. Numerous studies have demonstrated that LYC is effective at preventing oxidative stress caused by certain mycotoxins [16,17]. A recent study suggested that LYC alleviated aflatoxin B1-induced PANoptosis in chicken hepatocytes by altering mitochondrial homeostasis. Although the beneficial effects of LYC have been widely reported, its potential role in attenuating DON-induced intestinal toxicity and the precise mechanisms involved have not yet been clarified.

Ferroptosis is a recently identified form of cell death characterized by iron dependence, which leads to oxidative membrane damage [18]. Ferroptosis results from a redox imbalance and dysregulated redox-active enzymes [19]. Ferroptosis stimulation can impair glutathione (GSH) peroxidase activity through diverse pathways, thereby weakening cellular antioxidant defenses, promoting lipid reactive oxygen species (ROS) accumulation, and triggering oxidative cell death [20]. Some studies have suggested that ferroptosis is significantly connected to numerous pathophysiological processes of multiple disorders, including kidney injury, tumors, and intestinal ischemia [21]. A recent report emphasized that the relationship between the intestinal microbiota and ferroptosis involves iron metabolism, redox homeostasis, and lipid peroxidation [22]. Emerging evidence indicates that mycotoxins can trigger ferroptosis by disrupting cellular redox homeostasis and iron metabolism, resulting in organ dysfunction [23]. However, the precise involvement of ferroptosis in the protective role of LYC in alleviating DON-induced intestinal toxicity remains unclear.

Mitochondria are the pivotal organelles responsible for various cellular activities, thereby maintaining cellular balance. As a form of nonstandard autophagy, mitophagy involves the cellular process of selectively removing damaged mitochondria through the autophagic pathway [24,25]. However, both inadequate mitophagy and excessive mitophagy can negatively affect cell survival. Overactivation of mitophagy results in excessive clearance of mitochondria, ultimately leading to cell death. Conversely, insufficient mitophagy hinders the elimination of impaired mitochondria, causing mitochondrial dysfunction and reduced cell viability [26]. A previous study revealed that DON disrupts mitochondrial function by decreasing membrane potential and increasing ROS generation, thereby modulating mitophagy [27]. Phosphoglycerate mutase family member 5 (PGAM5) is a mitochondrial serine/threonine phosphatase that is predominantly localized to the inner membrane of mitochondria and plays a role in regulating mitochondrial stress and the antioxidative response [28]. PGAM5 contributes to mitophagy by maintaining PTEN-induced kinase 1 (PINK1) stability and modulating the PINK1–PRKN pathway to promote the degradation of impaired mitochondria [29]. Furthermore, PGAM5 can regulate cellular redox homeostasis by interacting with the Keap1–Nrf2 complex, thereby influencing antioxidant defense mechanisms [30]. Nevertheless, it is still undetermined whether LYC alleviates DON-induced intestinal impairment through PGAM5-dependent mitophagy.

The intestine, being the largest area regularly interacting with the external environment, serves as a primary barrier protecting against pathogen invasion. The intestinal barrier is largely composed of tight junction (TJ) proteins, which are crucial for maintaining the structure and function integrity of the intestinal mucosa [31]. Numerous studies have showed that DON can lead to severe impairment of intestinal barrier integrity and disrupts the immune system [32,33]. Pigs are viewed as the species most vulnerable to the effects of DON toxicity and intestinal porcine epithelial cells (IPEC-J2 cells) are acknowledged as a superior cellular model for exploring the effects of the intestinal barrier. Accordingly, IPEC-J2 cells were chosen to investigate the protective effects of LYC against DON-induced intestinal toxicity and elucidate the underlying mechanisms. PGAM5 acts as a pivotal regulator of cellular metabolic and redox balance, enhancing mitophagy to preserve redox balance and protect against cell damage induced by oxidative stress [34]. However, whether PGAM5-mediated mitophagy contributes to the protective effects of LYC against DON-induced intestinal injury has yet to be elucidated. This study aimed to elucidate the role and mechanism through which PGAM5 in LYC mitigates DON-induced intestinal toxicity, suggesting the development of innovative approaches for public health protection.

## Results

### LYC mitigated DON-induced intestinal barrier dysfunction in IPEC-J2 cells

**Fig. 1.**
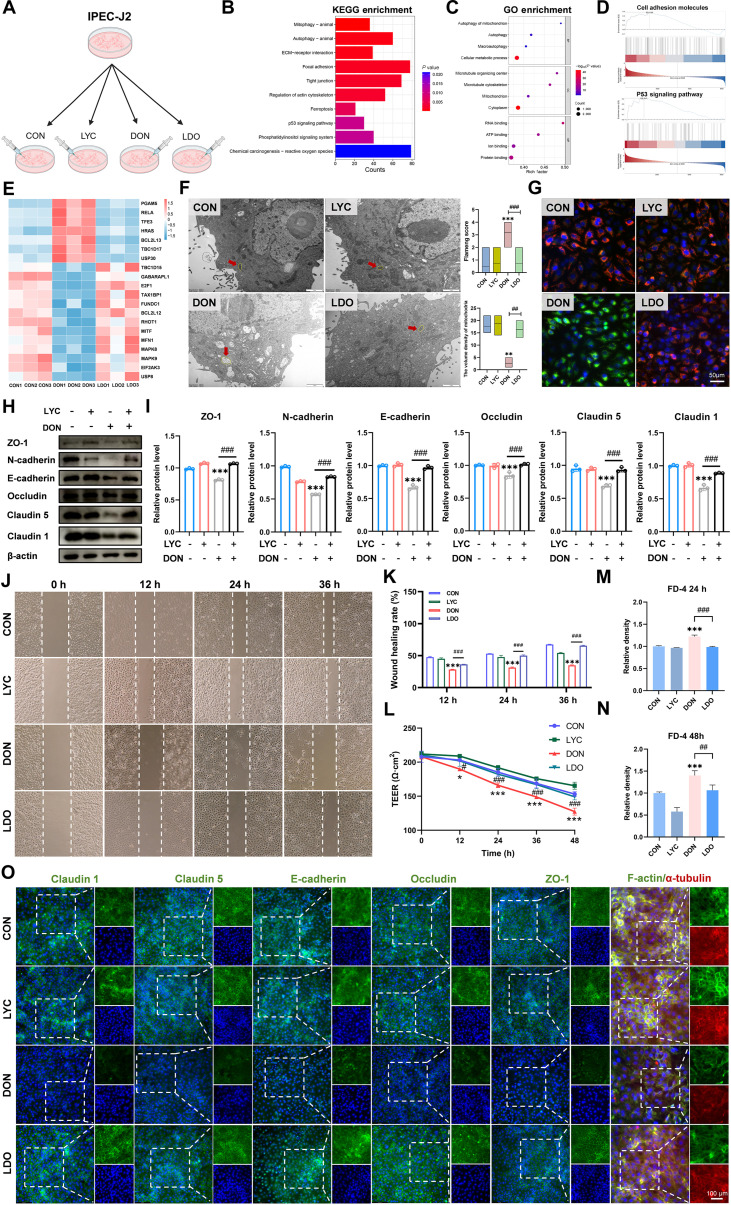
Lycopene (LYC) mitigated deoxynivalenol (DON)-induced intestinal barrier dysfunction of intestinal porcine epithelial (IPEC-J2) cells. (A) The IPEC-J2 cells were treated with control (CON), LYC, DON, and LDO. (B) Kyoto Encyclopedia of Genes and Genomes (KEGG) analysis. (C) Gene Ontology (GO) analysis. (D) Gene set enrichment analysis (GSEA). (E) Heatmap analysis. (F) Transmission electron microscopy (TEM) analysis. (G) Representative immunofluorescence (IF) images of JC-1. (H) Expression of tight junction (TJ)-related proteins. (I) Quantitation of the protein expression of TJ-related proteins. (J) Cell migration ability. (K) Wound healing rate. (L) Changes of transepithelial electrical resistance (TEER). (M) FITC-dextran 4 (FD-4) fluorescence intensity at 24 h. (N) FD-4 fluorescence intensity at 48 h. (O) Representative IF images of TJ-related proteins. Data are presented as the mean ± SD, *n* = 3. Symbol for the significance of differences between the CON group and the DON group: ^*^P < 0.05, ^**^P < 0.01, ^***^P < 0.001. Symbol for the significance of differences between the DON group and LDO group: ^#^*P* < 0.05, ^##^*P* < 0.01, ^###^*P* < 0.001.

To explore the protective role of LYC against DON-induced intestinal epithelial cell injury and elucidate its underlying mechanism, IPEC-J2 cells were individually treated with LYC and DON (Fig. 1A). First, we determined the cell viability of IPEC-J2 cells by a Cell Counting Kit-8 (CCK-8) assay and found that the cell viability decreased markedly after treatment with DON and increased markedly after treatment with LYC (Fig. S1A). We then performed RNA sequencing (RNA-seq) on IPEC-J2 cells. On the basis of the differentially expressed genes (DEGs) identified through sequencing, Kyoto Encyclopedia of Genes and Genomes (KEGG) analysis, Gene Ontology (GO) enrichment analysis, gene set enrichment analysis (GSEA), and heatmap analysis were conducted. KEGG analysis results indicated that the DEGs in the DON-treated group were enriched in pathways related to tight junctions (Fig. 1B). GO enrichment analysis showed that adenosine triphosphate binding was markedly enriched (Fig. 1C). GSEA revealed marked enrichment of genes associated with cell adhesion molecules following DON exposure (Fig. 1D). The heatmap analysis revealed that after DON treatment, the expressions of multiple genes related to mitophagy, autophagy, and cell death underwent marked changes (Fig. 1E). Given that disruption of tight junction and adhesion junction integrity is closely associated with epithelial barrier dysfunction [35], we then evaluated the protein expression levels of key TJ components. Western blot analysis revealed that DON reduced the protein expression levels of zonula occludens 1 (ZO-1), neural cadherin (N-cadherin), epithelial cadherin (E-cadherin), Occludin, Claudin-5, and Claudin-1, whereas LYC treatment effectively reversed these alterations (Fig. 1H and I). In line with the results of Western blot analysis, the results of immunofluorescence (IF) demonstrated that LYC attenuated the DON-induced decrease in the fluorescence intensity of ZO-1, Occludin, E-cadherin, Claudin-5, and Claudin-1 (Fig. 1O). Given that epithelial junctions are responsible for maintaining intestinal epithelial integrity and regulating paracellular permeability, we further evaluated barrier function by measuring the transepithelial electrical resistance (TEER) and FITC-dextran 4 (FD-4) permeability. Our findings indicated that compared with DON treatment, LYC treatment markedly increased the TEER and reduced FD-4 flux, indicating that LYC preserved epithelial barrier integrity and permeability (Fig. 1L to N). We subsequently conducted a scratch assay to evaluate the role of LYC in cell migration induced by DON. We found that LYC alleviated the DON-induced reduction in cell migration (Fig. 1J and K). KEGG enrichment analysis indicated that DON exposure predominantly affected genes associated with the regulation of the actin cytoskeleton after DON treatment. Furthermore, GO analysis revealed that genes associated with the microtubule cytoskeleton and microtubule organizing center were obviously enriched in the DON group. Similarly, the cytoskeleton and microtubule structures exhibited disorganization, along with diminished F-actin and α-tubulin signals. Notably, LYC treatment alleviated these cytoskeletal abnormalities, highlighting its protective role against DON-induced structural disruption (Fig. 1O). In summary, our results suggest that LYC mitigates the DON-induced disruption of TJs, cytoskeletal integrity, and barrier function.

### LYC inhibited DON-induced ferroptosis in IPEC-J2 cells

**Fig. 2.**
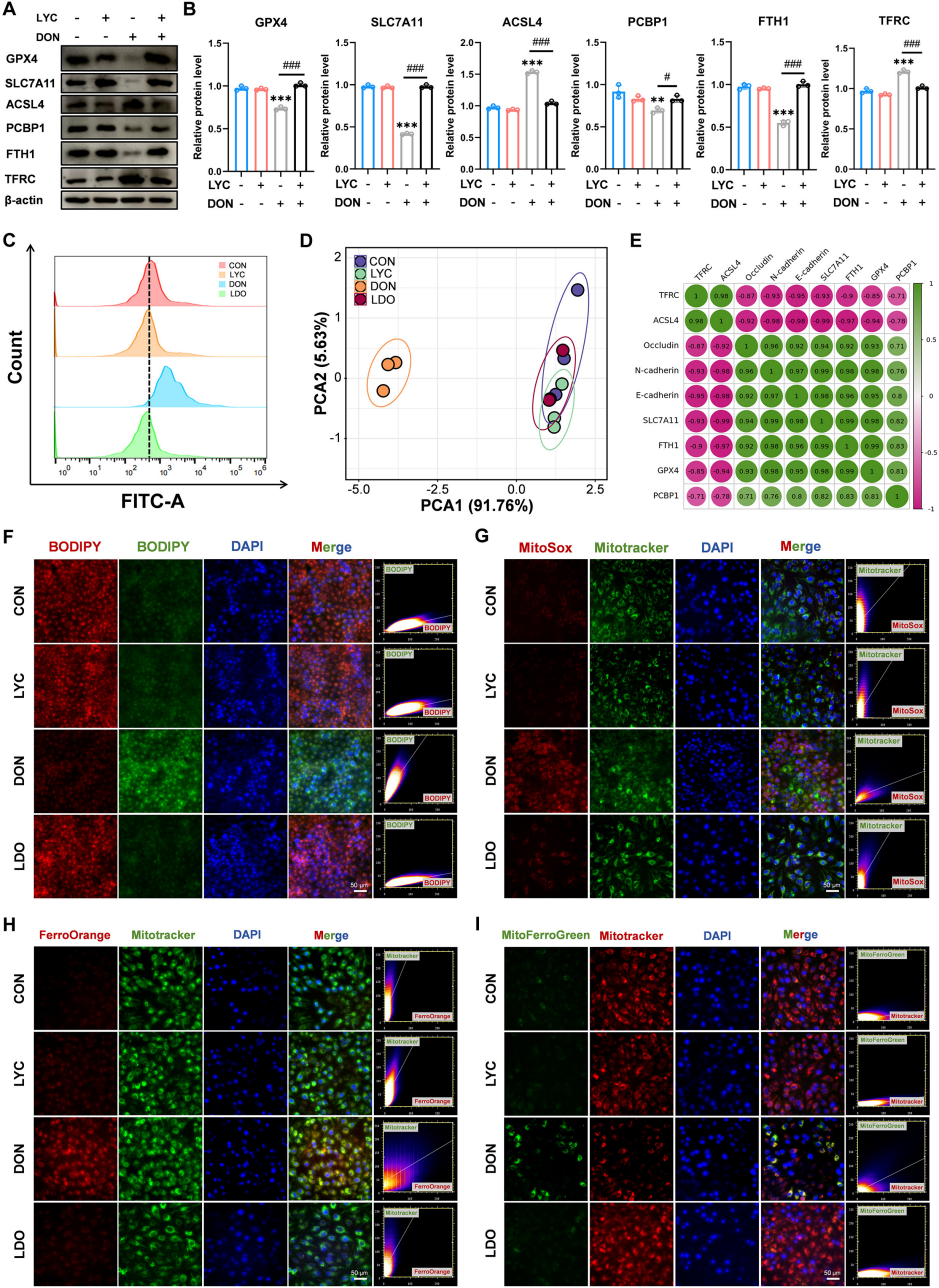
LYC inhibited DON-induced ferroptosis in IPEC-J2 cells. (A) Expression of ferroptosis-related proteins. (B) Quantitation of the protein expression of ferroptosis-related proteins. (C) Reactive oxygen species (ROS) level. (D) Principal component analysis (PCA). (E) Correlation analysis. (F) Lipid ROS level. (G) Mitochondrial ROS level. (H) Intracellular iron level. (I) Mitochondrial iron level. Data are presented as the mean ± SD, *n* = 3. Symbol for the significance of differences between the CON group and the DON group: ^**^P < 0.01, ^***^P < 0.001. Symbol for the significance of differences between the DON group and LDO group: ^#^*P* < 0.05, ^###^*P* < 0.001.

Accumulating studies have highlighted the strong association between mitochondrial homeostasis and the integrity of the intestinal epithelial barrier [36]. Mitochondrial dysfunction can compromise TJ organization and enhance epithelial permeability, whereas sustaining mitochondrial activity is essential for preserving intestinal barrier stability [37]. To verify whether the DON-induced disorder of the intestinal barrier is related to mitochondrial damage, we examined structural and functional alterations in mitochondria. The DON-treated group exhibited evident mitochondrial swelling, vacuolization, and cristae disruption, accompanied by increased Flameng scores. However, LYC effectively alleviated these alterations (Fig. 1F). Moreover, we utilized JC-1 staining to evaluate mitochondrial function and found that LYC restored the DON-induced reduction of the mitochondrial membrane potential (MMP) (Fig. 1G). Mitochondrial dysfunction leads to oxidative stress and energy metabolism disorders [38]. KEGG pathway analysis also revealed that the DEGs were enriched primarily in pathways such as chemical carcinogenesis-ROS after DON exposure. Excessive ROS generation is a key contributor to oxidative stress and results in mitochondrial dysfunction [39]. Consistently, our findings revealed a marked increase in ROS levels after DON treatment, but LYC effectively mitigated this increase in ROS levels (Fig. 2C). Ferroptosis is characterized by excessive accumulation of lipid-derived ROS, accompanied by mitochondrial damage. To clarify whether ferroptosis is involved in the protective role of LYC against DON-induced intestinal toxicity, we performed a series of ferroptosis-related assays. KEGG analysis also revealed that the DEGs were predominantly enriched in the ferroptosis pathway after DON treatment. Ferroptosis has been identified as a major factor involved in inflammatory responses and intestinal epithelial injury [40]. Consistent with the results of this transcriptomic analysis, Western blot analysis further revealed increased protein expression levels of acyl-CoA synthetase long chain family member 4 (ACSL4) and transferrin receptor (TFRC), which were accompanied by decreased protein expression levels of glutathione peroxidase 4 (GPX4), solute carrier family 7 member 11 (SLC7A11), poly (rC) binding protein 1 (PCBP1), and ferritin heavy chain 1 (FTH1) in the DON-treated group. However, LYC suppressed these alterations (Fig. 2A and B). The results revealed that DON treatment markedly reduced intracellular GSH levels, whereas LYC treatment effectively reversed the DON-induced decrease in GSH content (Fig. S1B). Ferroptosis is a unique process that occurs through the buildup of lipid peroxidation in an iron-dependent manner [41]. Therefore, we detected the level of ferrous iron using FerroOrange and Mito-FerroGreen probes. LYC markedly reversed the DON-induced increase in the fluorescence intensity of both probes, indicating the restoration of ferrous ion homeostasis (Fig. 2H and I). Moreover, DON exposure markedly increased lipid peroxidation and mitochondrial ROS levels, but LYC treatment reduced these changes (Fig. 2F and G). Principal component analysis of ferroptosis-related indicators further confirmed that LYC attenuated the alterations in ferroptosis-associated parameters induced by DON exposure (Fig. 2D). Afterward, correlation analysis revealed a strong connection between ferroptosis and TJs (Fig. 2E). Taken together, these findings indicate that LYC restores intestinal barrier function by inhibiting ferroptosis.

### LYC inhibited DON-induced excessive mitophagy in IPEC-J2 cells

**Fig. 3.**
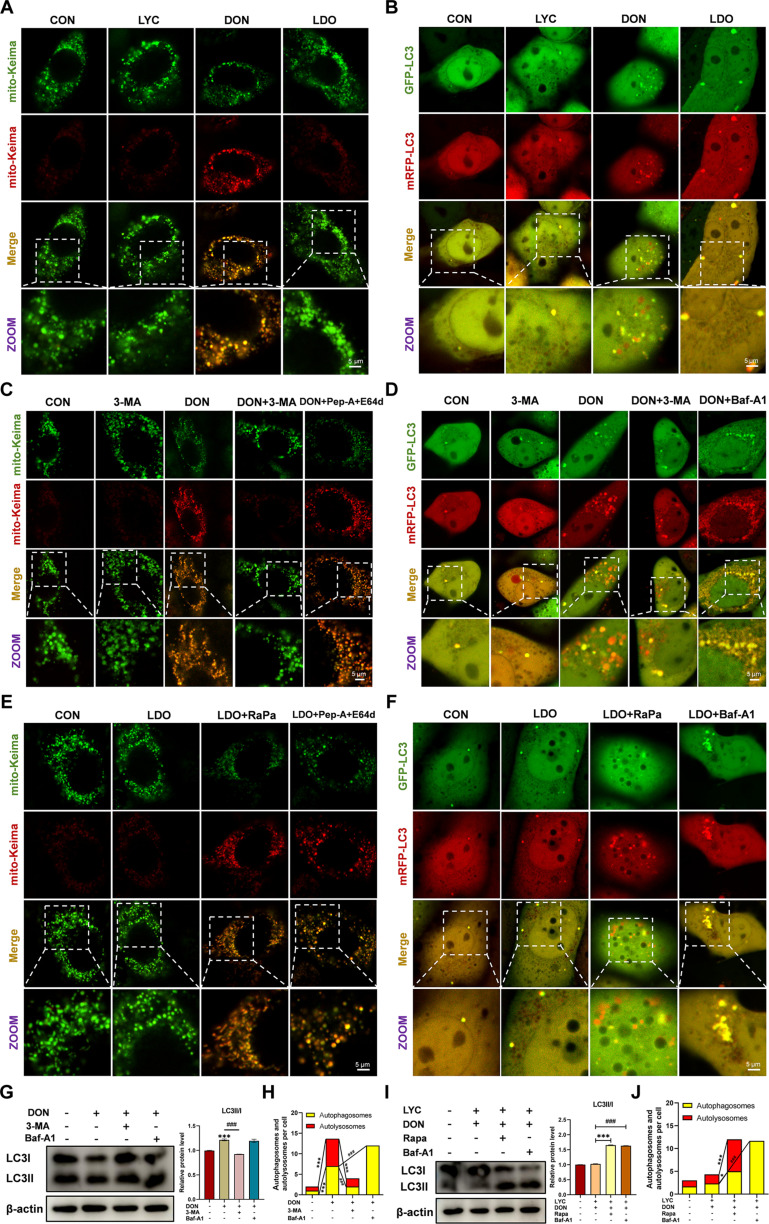
LYC inhibited DON-induced excessive mitophagy in IPEC-J2 cells. (A, C, and E) mito-Keima adenovirus transfection. (B, D, and F) mRFP-GFP-LC3 adenovirus transfection. (G) Western blot analysis of LC3II/I protein expression under 3-MA or Baf-A1 treatment. (H) Quantification of autophagosomes and autolysosomes under 3-MA or Baf-A1 treatment. (I) Western blot analysis of LC3II/I protein expression under RaPa or Baf-A1 treatment. (J) Quantification of autophagosomes and autolysosomes under RaPa or Baf-A1 treatment. Data are presented as the mean ± SD, *n* = 3. Symbol for the significance of differences between the CON group and the DON group, or the LDO group and LDO + RaPa group: ^*^P < 0.05 ^***^P < 0.001. Symbol for the significance of differences between the DON group and DON + 3-MA group, the DON group and DON + Baf-A1 group, or the LDO group and LDO + Baf-A1 group: ^###^*P* < 0.001.

Previous studies have demonstrated that mitophagy injury triggers intestinal inflammation and oxidative stress and harms the intestinal barrier [42]. Interestingly, KEGG analyses revealed enrichment of genes associated with mitophagy and autophagy in animals following DON exposure. Consistent with these findings, the results of the GO enrichment analysis revealed that the DEGs were distributed mainly in pathways related to mitophagy, autophagy, and macroautophagy. Increasing evidence indicates that mitophagy and ferroptosis closely interact, with mitophagy processes driving ferroptosis via the degradation of anti-ferroptotic factors and the promotion of iron-mediated lipid peroxidation and ROS generation [43]. To investigate whether LYC mitigates DON-induced autophagy, we measured key autophagy biomarkers. Western blot analysis revealed that LYC attenuated the DON-induced up-regulation of Parkinson juvenile disease protein 2 (PARKIN), PINK1, and LC3II/I protein expression and the down-regulation of P62 and translocase of outer mitochondrial membrane 20 (TOMM20) protein expression. Subsequently, mitophagy activity was further assessed using a mito-Keima probe assay. We found that DON led to higher levels of red fluorescence and lower levels of green fluorescence (Fig. 3A and Fig. S1C). Furthermore, 3-methyladenine (3-MA) suppressed red fluorescence following DON exposure, confirming the restoration of mitophagic flux. Since baflomycin-A1 (Baf-A1) acts as a late-stage autophagy inhibitor by preventing the fusion of autophagosomes and interferes with mito-Keima detection, Pep-A and E64d were used to assess mitophagic flux. The results revealed an increased intensity of acidic puncta labeled by the mito-Keima fluorescent protein after Pep-A and E64d treatment (Fig. 3C and Fig. S3E). In addition, LYC reversed the DON-induced increase in mitochondrial autophagic flux.

Interestingly, when rapamycin (RaPa) or Pep-A + E64d was applied, the ability of LYC to mitigate the DON-induced increase in autophagic flux was abolished (Fig. 3E and Fig. S3F). Moreover, we assessed autophagic flux in IPEC-J2 cells through transient transfection with mRFP-GFP-LC3 followed by a fluorescence assay. Since the green fluorescent protein (GFP) signal was quenched in the acidic environment of lysosomes, only puncta with monomeric red fluorescent protein (mRFP) (red) without GFP fluorescence were autolysosomes, and yellow puncta with both GFP and mRFP fluorescence corresponded to autophagosomes. We observed that DON exposure led to an increase in both yellow and red puncta, whereas LYC effectively attenuated DON-induced accumulation of GFP and RFP positive puncta, highlighting its protective effect on autophagic activity (Fig. 3B). To distinguish between enhanced autophagosome formation and impaired degradation, we further examined the effects of DON in the presence of the autophagy inhibitors. The results indicated that DON reduced LC3II/I levels when autophagosome formation was blocked by 3-MA, but increased LC3II/I levels when autophagosome degradation was suppressed by Baf-A1 (Fig. 3G). Similarly, DON increased the number of yellow puncta and decreased the number of red puncta after Baf-A1 treatment, whereas both yellow and red puncta were reduced after 3-MA treatment (Fig. 3D and H). However, the protective effects of LYC were abolished when the cells were cotreated with RaPa. After Baf-A1 treatment, LYC treatment resulted in an increase in yellow puncta and a decrease in red puncta (Fig. 3F and J). The corresponding alterations in LC3 protein levels aligned with these findings (Fig. 3I). These findings indicate that DON primarily enhances autophagy initiation rather than affecting autophagosome degradation at later stages. More importantly, these results demonstrate that LYC effectively restored the disruption of mitophagy homeostasis caused by DON exposure, thereby modulating excessive mitophagy.

### LYC alleviated the DON-induced up-regulation of PGAM5 protein expression in IPEC-J2 cells

**Fig. 4.**
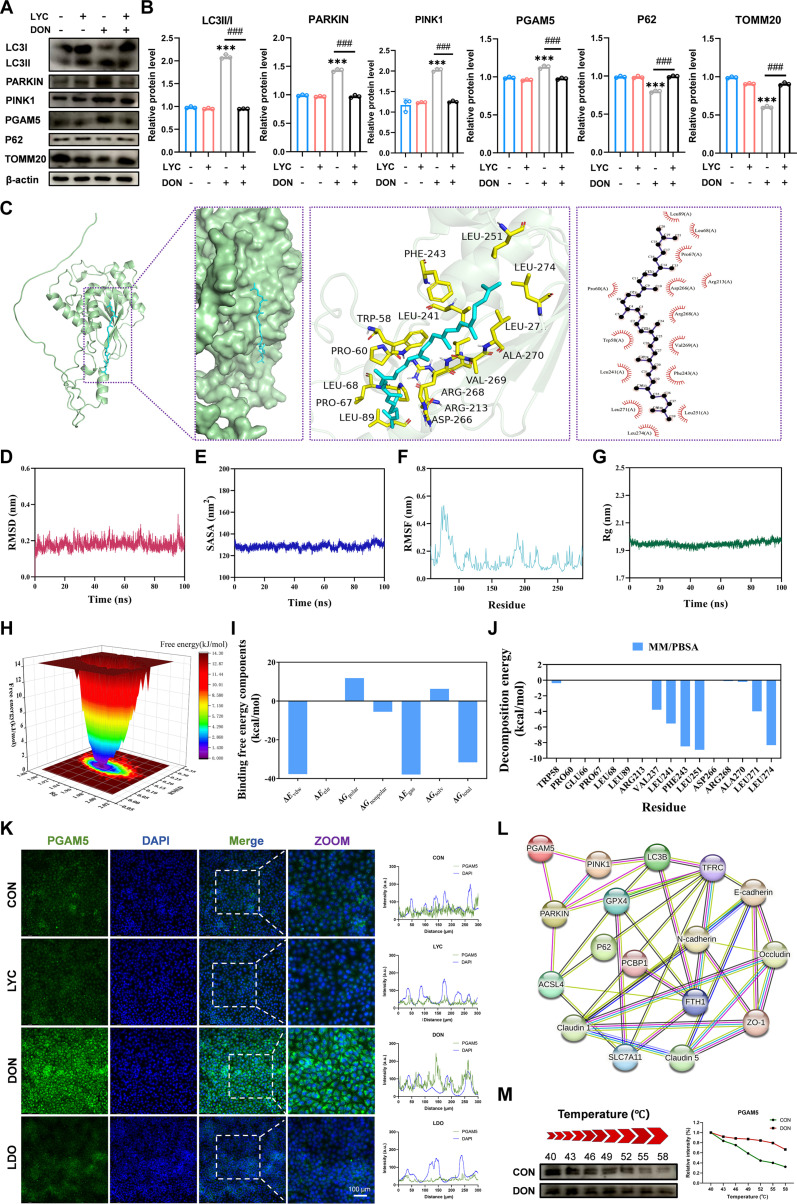
LYC alleviated the DON-induced up-regulation of phosphoglycerate mutase family member 5 (PGAM5) protein expression in IPEC-J2 cells. (A) Expression of mitophagy-related proteins. (B) Quantitation of the protein expression of mitophagy-related proteins. (C) Molecular docking simulation for the ligand–protein binding of LYC with PGAM5. (D) Root mean square deviation (RMSD) of the complex. (E) Solvent accessible surface area (SASA). (F) Root mean square fluctuation (RMSF) number. (G) Radius of gyration number. (H) The Gibbs energy landscape of complex. (I) Binding free energy components. (J) Decomposition energy. (K) Representative IF images of PGAM5. (L) protein–protein interaction (PPI) network. (M) Thermal stability analysis of PGAM5. Data are presented as the mean ± SD, *n* = 3. Symbol for the significance of differences between the CON group and the DON group: ^***^P < 0.001. Symbol for the significance of differences between the DON group and LDO group: ^###^*P* < 0.001.

PGAM5 is known to control autophagic processes by mediating the generation of autophagosomes and plays a direct role in regulating mitophagy through the PINK1 pathway [29]. A heatmap was used to illustrate the expression profiles of key genes involved in the enriched pathways, revealing a marked up-regulation of PGAM5 expression following DON exposure, which was reversed by LYC treatment. The protein expression levels of PGAM5 tended to increase, as determined by Western blot analysis, in the DON treatment group, whereas LYC restored these levels (Fig. 4A and B). Consistently, IF analysis further confirmed these results, revealing changes in the distribution of PGAM5 (Fig. 4K). In order to understand the possible binding interactions between LYC and DON and PGAM5, we performed molecular docking and dynamics simulations, respectively. The docking results revealed a stable interaction between DON and PGAM5 (Fig. S1F). Furthermore, a molecular dynamics simulation further validated the structural stability of the DON and PGAM5 complex (Fig. S1G to M). The docking results demonstrated that LYC could form a stable binding conformation with PGAM5 (Fig. 4C). Furthermore, the results of molecular dynamics simulations further confirmed the stability of the LYC–PGAM5 interaction. The results showed that the trajectory of the LYC–PGAM5 complex remained stable throughout the simulation period, with relatively small fluctuations, indicating a stable interaction between LYC and PGAM5 (Fig. 4D to J). According to the cellular thermal shift assay (CETSA) results, DON increased the thermal stability of PGAM5, highlighting the interaction of DON and PGAM5 (Fig. 4M). We subsequently constructed a protein–protein interaction network diagram, which revealed the close interactions among PGAM5, ferroptosis pathway proteins, and TJ proteins (Fig. 4L). Therefore, we hypothesize that LYC attenuates DON-induced intestinal barrier dysfunction by suppressing PGAM5-mediated mitophagy, which in turn inhibits ferroptosis and contributes to the restoration of intestinal barrier integrity.

### PGAM5 overexpression counteracted the protective effects of LYC against DON-induced excessive autophagy

**Fig. 5.**
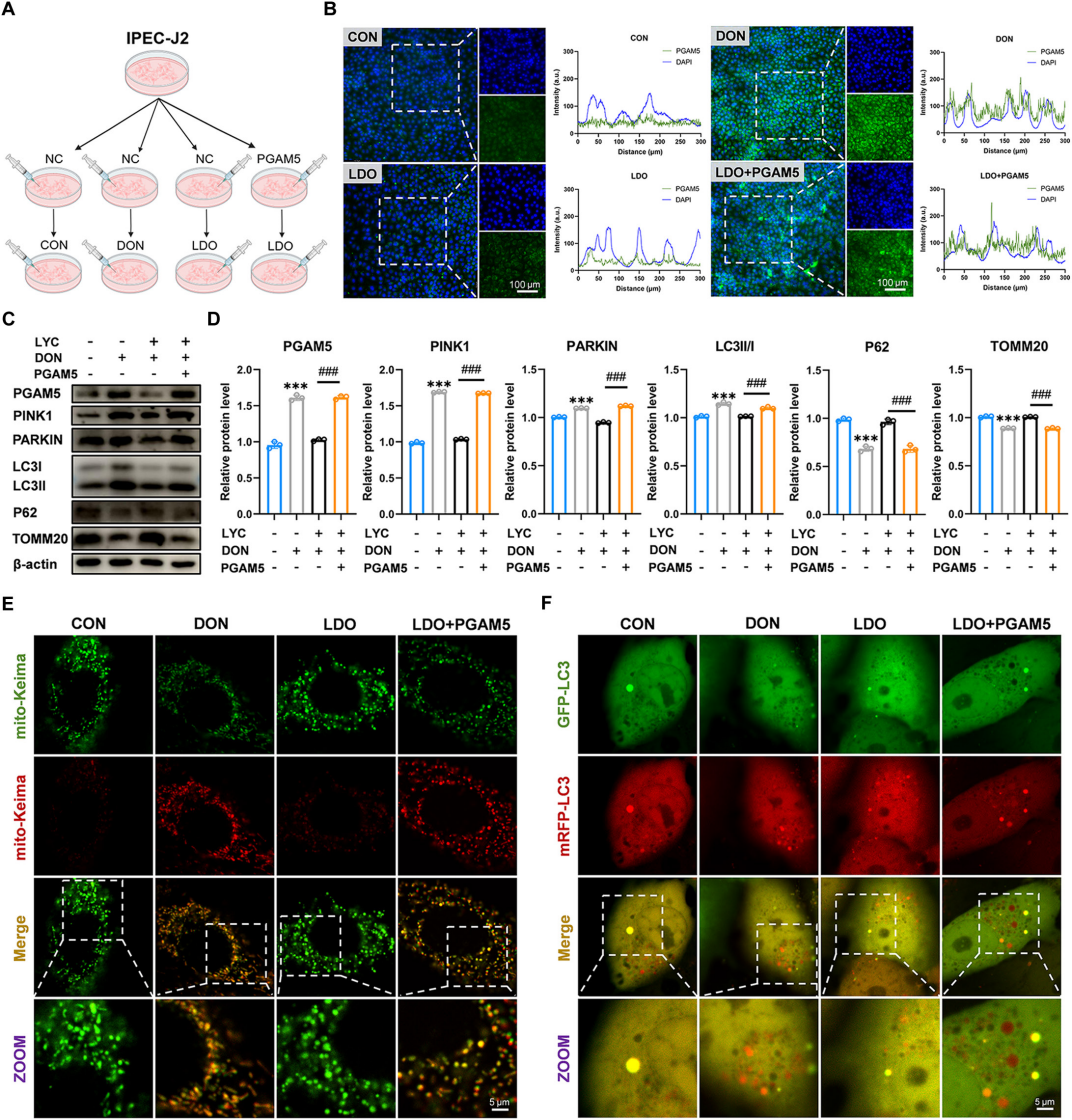
PGAM5 overexpression counteracted the protective effects of LYC against DON-induced excessive autophagy. (A) The IPEC-J2 cells were treated with CON, DON, LDO, and LDO + PGAM5. (B) Representative IF images of PGAM5. (C) Expression of mitophagy-related proteins. (D) Quantitation of the protein expression of mitophagy-related proteins. (E) mito-Keima adenovirus transfection. (F) mRFP-GFP-LC3 adenovirus transfection. Data are presented as the mean ± SD, *n* = 3. Symbol for the significance of differences between the CON group and the DON group: ^***^P < 0.001. Symbol for the significance of differences between the LDO group and LDO + PGAM5 group: ^###^*P* < 0.001.

To demonstrate the potential role of PGAM5 in the ability of LYC to relieve DON-induced intestinal injury, we carried out PGAM5 overexpression experiments (Fig. 5A and Fig. S1D). We found that PGAM5 overexpression up-regulated the protein levels of PGAM5, PINK1, LC3II/I, and PARKIN, which was accompanied by decreases in the protein levels of P62 and TOMM20 (Fig. 5C and D). Notably, IF analysis confirmed the trends of PGAM5 expression observed by Western blot analysis (Fig. 5B). Moreover, compared with the LDO group, the groups in which DON and PGAM5 were overexpressed presented pronounced increases in both red and yellow puncta, indicating increased autophagic activity (Fig. 5F). Furthermore, the mito-Keima assay revealed that LYC failed to reverse the increase in red fluorescence intensity induced by DON under conditions of LYC and DON exposure combined with PGAM5 overexpression (Fig. 5E and Fig. S3C). Collectively, these findings indicate that LYC relieves DON-induced alterations in autophagy through the modulation of PGAM5 protein expression.

### PGAM5 overexpression eliminated the antagonistic effects of LYC on DON-induced ferroptosis

**Fig. 6.**
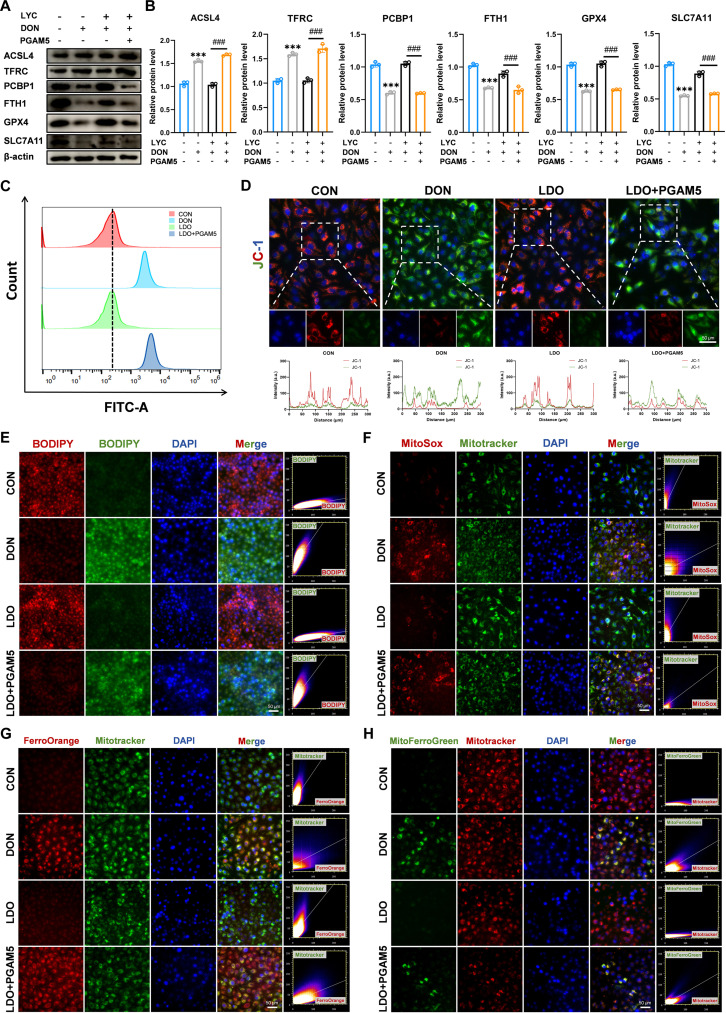
PGAM5 overexpression eliminated the antagonistic effects of LYC on DON-induced ferroptosis. (A) Expression of ferroptosis-related proteins. (B) Quantitation of the protein expression of ferroptosis-related proteins. (C) ROS level. (D) Representative IF images of JC-1. (E) Lipid ROS level. (F) Mitochondrial ROS level. (G) Intracellular iron level. (H) Mitochondrial iron level. Data are presented as the mean ± SD, *n* = 3. Symbol for the significance of differences between the CON group and the DON group: ^***^P < 0.001. Symbol for the significance of differences between the LDO group and LDO + PGAM5 group: ^###^*P* < 0.001.

PGAM5 exerts a protective effect against ferroptosis by inhibiting lipid peroxidation and limiting the intracellular accumulation of ferric ions [44]. To further determine whether the ability of LYC to alleviate DON-induced ferroptosis is linked to PGAM5, we examined the expression of ferroptosis-related markers. Western blot analysis demonstrated that PGAM5 overexpression suppressed the ability of LYC to mitigate DON-induced ferroptosis, including increases in ACSL4 and TFRC protein expression levels, and decreases in the PCBP1, FTH1, GPX4, and SLC7A11 protein expression levels (Fig. 6A and B). Consistent with these findings, we observed a marked increase in ROS levels after PGAM5 overexpression (Fig. S3A). Moreover, the total GSH (T-GSH) level was markedly lower in the LDO + PGAM5 group than in the LDO group (Fig. S3B). Subsequently, PGAM5 overexpression failed to prevent the DON-induced increase in ROS levels even in the presence of LYC (Fig. 6C). Notably, PGAM5 overexpression also resulted in a marked reduction in the MMP, further supporting its involvement in mitochondrial dysfunction in response to DON exposure (Fig. 6D). Given that mitochondria are the main producers of ROS, we also detected the level of ROS in mitochondria. These data indicated that PGAM5 overexpression increased the level of mitochondrial ROS (Fig. 6C). Consistent with these findings, we also found that PGAM5 overexpression prevented LYC from alleviating DON-induced lipid peroxidation (Fig. 6E and F). Since the disruption of the ferrous balance is a feature of ferroptosis, intracellular ferric ion levels were also quantified. The results showed that PGAM5 overexpression suppressed the restoration of iron homeostasis after LYC treatment (Fig. 6G and H). These findings demonstrate that LYC mitigates DON-induced ferroptosis by targeting the PGAM5 protein.

### PGAM5 overexpression abolished the protective effect of LYC on DON-induced impairment of intestinal barrier function

**Fig. 7.**
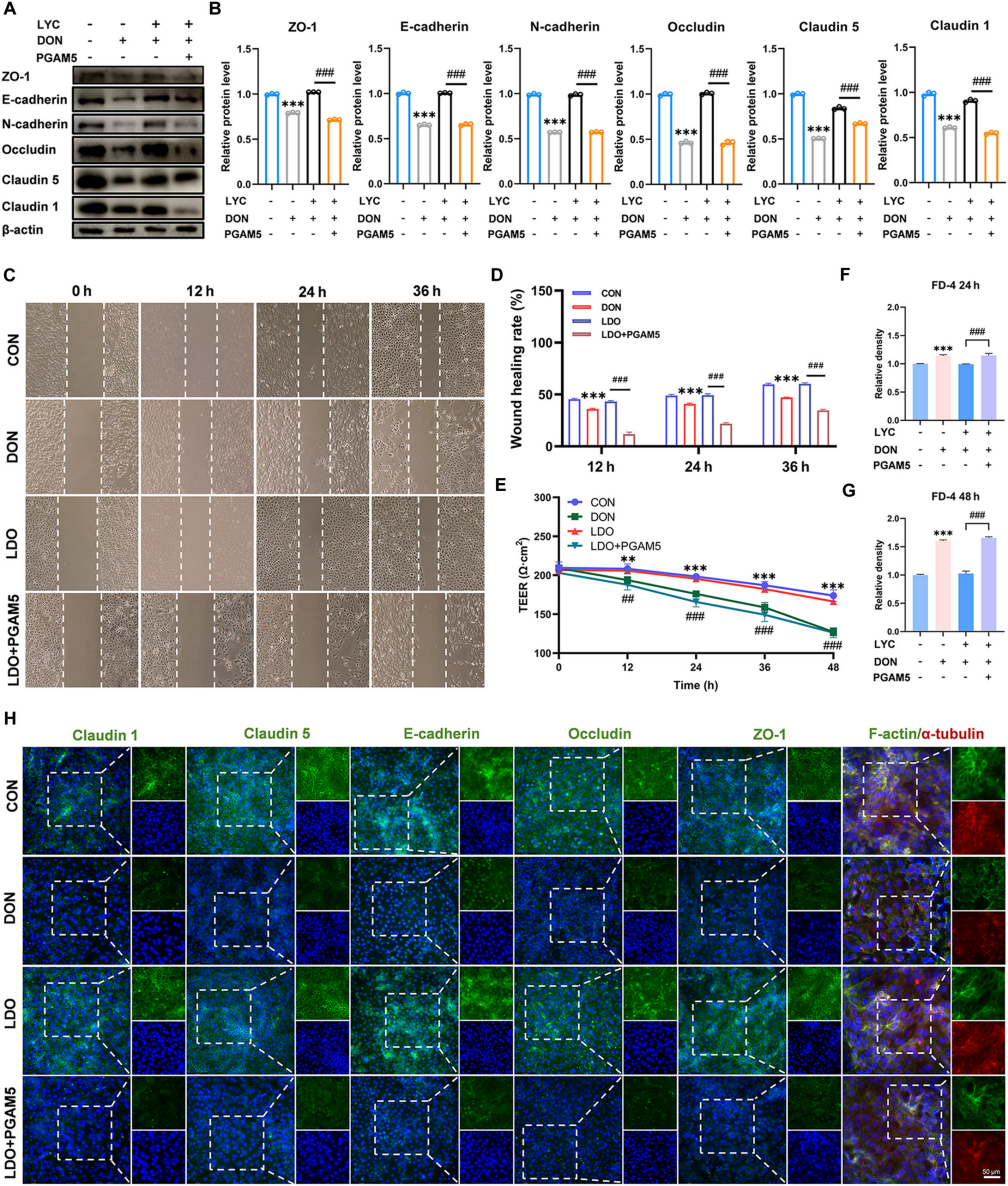
PGAM5 overexpression abolished the protective effect of LYC on DON-induced impairment of intestinal barrier function. (A) Expression of TJ-related proteins. (B) Quantitation of the protein expression of TJ-related proteins. (C) Cell migration ability. (D) Wound healing rate. (E) Changes of TEER. (F) FD-4 fluorescence intensity at 24 h. (G) FD-4 fluorescence intensity at 48 h. (H) Representative IF images of TJ-related proteins. Data are presented as the mean ± SD, *n* = 3. Symbol for the significance of differences between the CON group and the DON group: ^**^P < 0.01, ^***^P < 0.001. Symbol for the significance of differences between the LDO group and LDO + PGAM5 group: ^##^*P* < 0.01, ^###^*P* < 0.001.

To further define the effect of PGAM5 on the ability of LYC to regulate DON-induced intestinal barrier damage, we assayed the levels of proteins related to barrier function after PGAM5 overexpression. The protein expression levels of ZO-1, N-cadherin, Occludin, E-cadherin, Claudin-5, and Claudin-1 were markedly reduced (Fig. 7A and B). The IF results were consistent with those of the Western blot analysis, and the protein expression levels of Claudin-1, Claudin-5, E-cadherin, Occludin, and ZO-1 decreased after PGAM5 overexpression (Fig. 7H). Notably, our findings verified that LYC alleviated DON-induced disruption of the cell cytoskeleton and the disorganized F-actin structure (Fig. 7H). PGAM5 overexpression also counteracted the ability of LYC to reverse the DON-induced increase in FD-4 permeability and the decrease in the TEER (Fig. 7E to G). Moreover, PGAM5 overexpression also abolished the ability of LYC to mitigate the DON-induced inhibition of cell migration (Fig. 7C and D). Taken together, our findings indicate that LYC prevents DON-induced injury of intestinal barrier function through regulation of PGAM5 protein expression.

### PGAM5 knockdown alleviated DON-induced impairment of intestinal barrier function by inhibiting ferroptosis and autophagy

To further evaluate whether PGAM5 is essential for DON-induced intestinal epithelial injury, we performed PGAM5 loss-of-function experiments (Fig. S1E). Our study revealed that PGAM5 knockdown markedly restored the MMP, as evidenced by JC-1 staining (Fig. S2B). IF assays demonstrated that PGAM5 knockdown attenuated the DON-induced decrease in the fluorescence intensity of Occludin, E-cadherin, and Claudin-1, indicating improved intestinal epithelial barrier integrity (Fig. S2A). Furthermore, PGAM5 knockdown attenuated the excessive DON-induced autophagy and mitophagy, as indicated by decreased LC3 puncta formation and mito-Keima red fluorescence (Fig. S2C and D and Fig. S3D). BODIPY and FerroOrange staining revealed that PGAM5 knockdown markedly attenuated DON-induced lipid peroxidation and intracellular ferrous iron accumulation (Fig. S2E and F). Collectively, these findings demonstrated that PGAM5 knockdown effectively alleviated DON-induced mitochondrial dysfunction, excessive mitophagy, ferroptosis, and epithelial barrier injury.

### LYC relieved DON-induced intestinal barrier injury by inhibiting PGAM5-mediated mitophagy-dependent ferroptosis

**Fig. 8.**
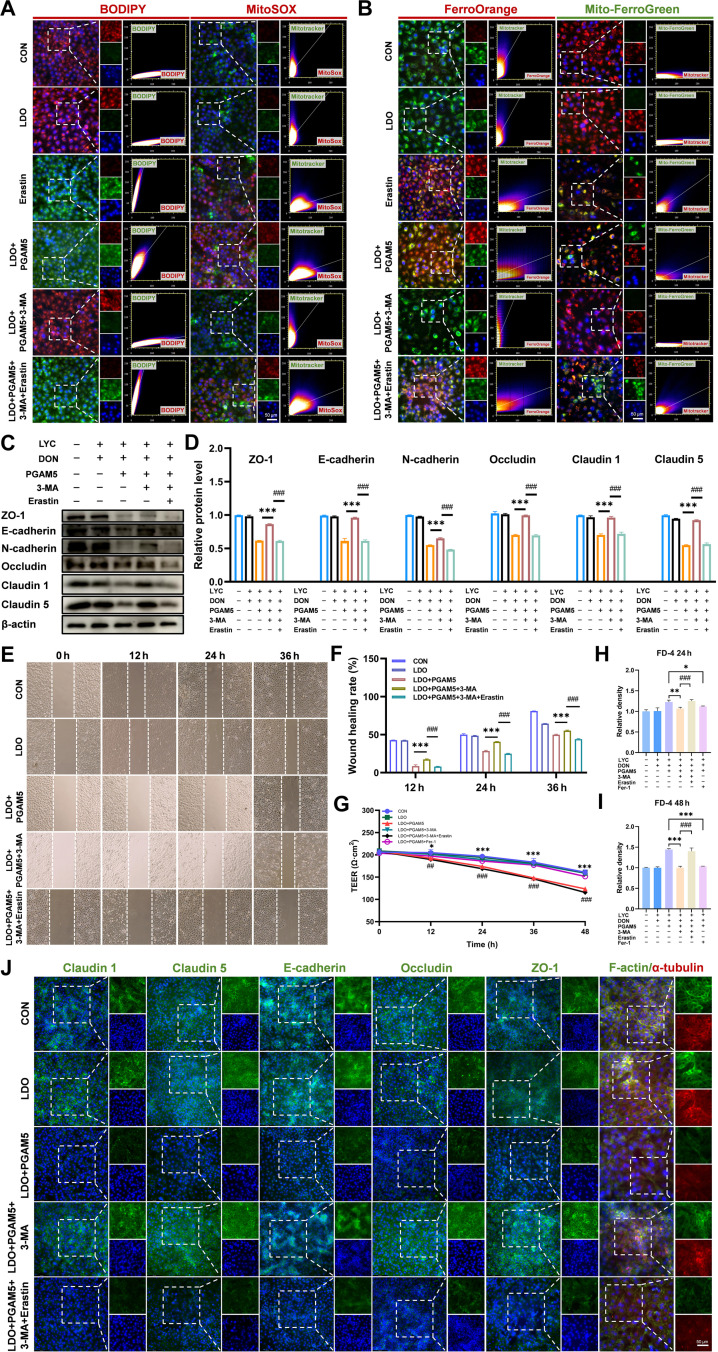
LYC relieved DON-induced intestinal barrier injury by inhibiting PGAM5-mediated mitophagy-dependent ferroptosis. (A) Lipid ROS level and mitochondrial ROS level. (B) Intracellular iron level and mitochondrial iron level. (C) Expression of TJ-related proteins. (D) Quantitation of the protein expression of TJ-related proteins. (E) Cell migration ability. (F) Wound healing rate. (G) Changes of TEER. (H) FD-4 fluorescence intensity at 24 h. (I) FD-4 fluorescence intensity at 48 h. (J) Representative IF images of TJ-related proteins. Data are presented as the mean ± SD, *n* = 3. Symbol for the significance of differences between the LDO + PGAM5 group and the LDO + PGAM5 + 3-MA group or the LDO + PGAM5 + Fer-1 group: ^*^P < 0.05, ^**^P < 0.01, ^***^P < 0.001. Symbol for the significance of differences between the LDO + PGAM5 + 3-MA group and the LDO + PGAM5 + 3-MA + Erastin group: ^##^*P* < 0.01, ^###^*P* < 0.001.

To further verify whether LYC relieves DON-induced intestinal barrier injury in connection with PGAM5-mediated autophagy and ferroptosis, IPEC-J2 cells were treated with an autophagy inhibitor (3-MA) and a ferroptosis inducer (Erastin). According to the results of fluorescence staining with BODIPY and MitoSOX, 3-MA markedly attenuated lipid peroxidation and mitochondrial ROS accumulation, whereas Erastin abolished these protective effects (Fig. 8A).

**Fig. 9.**
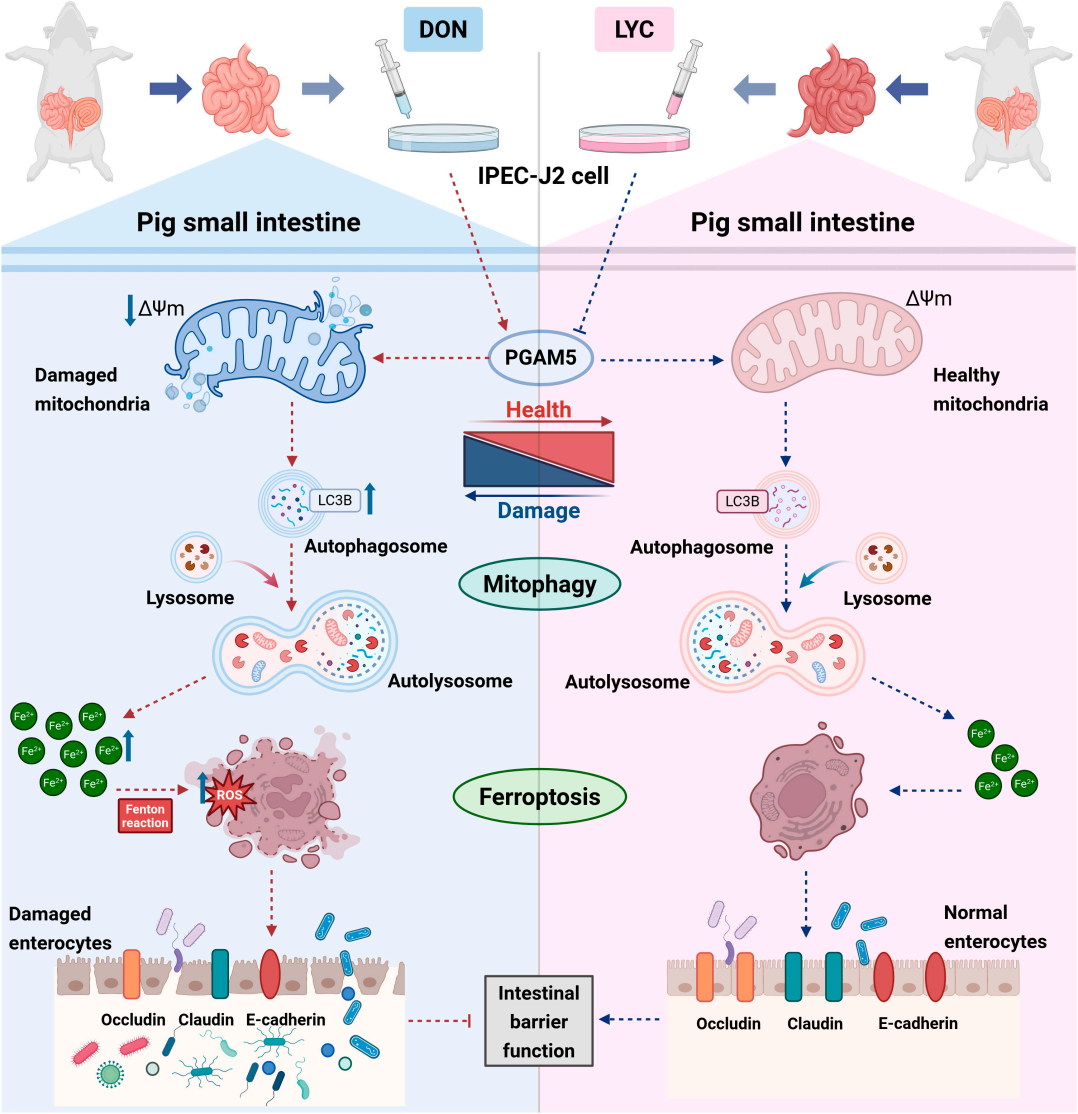
LYC alleviated DON-induced structural disruption of porcine intestinal epithelial barrier functions by targeting the PGAM5-mediated mitophagy-dependent ferroptosis.

Consistent with these findings, FerroOrange and Mito-FerroGreen staining indicated a decrease in intracellular and mitochondrial iron overload after 3-MA treatment, but Erastin reversed these changes (Fig. 8B). We also demonstrated a marked decrease in ROS levels after 3-MA treatment, but Erastin treatment increased ROS levels (Fig. S3A). In addition, the inhibition of autophagy with 3-MA markedly restored the intracellular GSH level after PGAM5 overexpression, whereas treatment with Erastin further exacerbated the decrease in the GSH level (Fig. S3B). We subsequently assessed barrier function in IPEC-J2 cells. Western blot and IF analysis further revealed that 3-MA restored the down-regulation of TJ protein (ZO-1, E-cadherin, N-cadherin, Occludin, Claudin-1, and Claudin-5) expression levels, whereas Erastin exacerbated the impairment of barrier-associated protein expression levels (Fig. 8C, D, and J). Interestingly, we observed that the F-actin cytoskeleton was systematically organized upon 3-MA treatment, but Erastin disrupted these structures (Fig. 8J). The wound-healing assay revealed that the decrease in cell migration was markedly reversed in the presence of 3-MA, whereas Erastin treatment further exacerbated the suppression of cell migration (Fig. 8E and F). Similarly, TEER and FD-4 assays demonstrated that 3-MA and ferrostatin-1 (Fer-1) maintained barrier integrity, whereas Erastin exacerbated these impairments (Fig. 8G to I). Collectively, these findings indicate that LYC protects against DON-induced intestinal barrier injury by inhibiting PGAM5-mediated mitophagy-dependent ferroptosis (Fig. 9).

## Discussion

DON, a highly prevalent and agriculturally important mycotoxin, represents a major threat to global food security owing to its extensive contamination and toxicity to humans [45]. Previous reports have suggested that DON can disrupt the imbalance in intestinal microecology, leading to impaired barrier integrity and compromised host immune function [46]. Therefore, it is crucial to study the toxicity mechanism of DON on intestinal and find effective strategies to alleviate the adverse effects caused by DON. LYC, as a natural, potent antioxidant, is vital for safeguarding cells against oxidative damage [47]. In this study, we found that LYC alleviated DON-induced intestinal porcine epithelial barrier structure and function damage. Of note, our results demonstrated that LYC effectively ameliorated DON-induced characteristic mitochondrial morphological changes of ferroptosis, accompanied by massive autophagic vesicles in the IPEC-J2 cells. We also found that LYC mitigated DON-induced excessive mitophagy and ferroptosis. Interestingly, we identified PGAM5 as a critical regulatory molecule that links excessive mitophagy to ferroptosis. We also report the pivotal role for PGAM5 in the regulation of intestinal structure and barrier function. Notably, there is a marked increase in PGAM5 expression after DON treatment, but LYC reversed it. PGAM5 knockdown alleviated DON-induced impairment of intestinal barrier function by inhibiting ferroptosis and mitophagy. Importantly, PGAM5 overexpression abolished the protective effects of LYC against DON-induced intestinal barrier impairment through the regulation of excessive mitophagy-dependent ferroptosis. This phenotype appears to be primarily driven by PGAM5’s direct regulation of genes involved in mitophagy-dependent ferroptosis during intestinal barrier impairment, as indicated by intervening with autophagy or ferroptosis inhibitor. We further demonstrated that LYC targeted the PGAM5, resulting in down-regulation of PGAM5 levels, which, in turn, eliminated excessive mitophagy and thus inhibited ferroptosis, eventually alleviating DON-induced disruption of porcine intestinal epithelial barrier functions. We innovatively discover that LYC restored DON-induced impairment of the porcine intestinal barrier through the inhibition of PGAM5-mediated mitophagy-dependent ferroptosis. Together, these findings highlight that PGAM5 serves as a promising therapeutic target for alleviating DON-induced intestinal epithelial injury and provides new perspectives for developing strategies to treat intestinal diseases associated with mycotoxin exposure.

The intestinal system is regularly regarded as the most marked interface between the body and the external environment, constituting the first line of defense against foodborne toxins [48]. The intestine serves as the primary site of DON absorption and the main target of its toxic effects [49]. Intestinal epithelial cells form a physical barrier through TJs and adherent junctions, protecting the intestine against microbiota and harmful substances [50]. Mycotoxins have detrimental effects on the intestinal epithelium, thereby compromising barrier integrity and disrupting gut microbial homeostasis [51]. Increasing evidence has demonstrated that DON exposure leads to pronounced intestinal toxicity, characterized by disruption of barrier function and impairment of epithelial integrity [52]. In agreement with our findings, DON exposure disrupted the integrity of TJ proteins, ultimately causing intestinal barrier impairment. LYC, a bright red carotenoid pigment, plays a pivotal role in maintaining intestinal homeostasis. LYC preserves epithelial barrier integrity and the function of TJs, thereby preventing intestinal injury [53]. In our study, we revealed that LYC effectively alleviated DON-induced intestinal injury by increasing the expression levels of TJ proteins, which contributed to maintaining intestinal epithelial integrity and preventing intestinal barrier dysfunction.

Ferroptosis refers to an adaptive cell death process that is induced by excessive lipid peroxidation and cellular metabolic activity [54]. Mitochondrial dysfunction has been suggested to be a key driver of ferroptosis through increases in ROS generation and oxidative stress [20,55]. In the present study, DON exposure induced marked alterations in mitochondrial morphology and function, which is consistent with previous evidence describing that revealed the distinctive mitochondrial features of ferroptosis [56]. Interestingly, our findings also indicated that DON treatment modulated the expression of ferroptosis-related markers, further confirming the activation of ferroptosis. Furthermore, ferroptosis has been identified as a key regulatory pathway, related to the development of multiple diseases, especially intestinal diseases [57]. Recent studies have shown that ferroptosis is related to the impairment of the intestinal epithelium through disturbance in the cellular redox balance [20]. Our results also revealed that DON exposure was clearly sufficient for evaluating ROS and ferrous ion levels in the cytosol and mitochondria, leading to enhanced lipid peroxidation and impaired intestinal barrier function. Notably, LYC is extensively known for its protective role in various pathological conditions associated with oxidative stress [58]. Emerging evidence has indicated that LYC mitigates mycotoxin-induced intestinal toxicity by modulating iron metabolism and preserving redox homeostasis [59]. We also revealed that LYC attenuated DON-induced ferroptosis, thereby restoring intestinal damage. Collectively, these findings highlight that the regulatory role of LYC in ferroptosis is pivotal in mitigating DON-induced intestinal barrier dysfunction.

Ferroptosis and mitophagy are closely interlinked processes that cooperatively modulate cellular redox homeostasis and iron metabolism [60,61]. Mitophagy is crucial for preserving both mitochondrial and cellular balance through the selective removal of damaged or superfluous mitochondria [62]. However, excessive mitophagy activation aggravates ferroptosis through the degradation of key cellular components and the consequent release of iron and ROS, thereby disrupting lipid metabolism and enhancing oxidative stress [63]. Some studies also validated that mitophagy is indispensable for intestinal health, where it contributes to the preservation of the epithelial barrier and intestinal homeostasis [42]. Recent reports have shown that mycotoxins cause mitochondrial injury, which is accompanied by increased PINK1/PARKIN-mediated mitophagy in hepatocytes [64]. Our results consistently proved that DON exposure promoted mitophagy activity and elevated autophagic flux. LYC exerts a protective effect by regulating abnormal mitophagy [65]. Recent research has shown that the suppression of autophagy is essential for the activation of TJ proteins induced by LYC treatment [66]. Our study also revealed that LYC mitigated intestinal barrier dysfunction primarily by inhibiting DON-induced ferroptosis and aberrant mitophagy, emphasizing the pivotal involvement of mitophagy in DON-induced intestinal toxicity.

PGAM5 is predominantly found inside the mitochondria and is related to controlling mitophagy, mitochondrial dynamics, and cell death [67]. PGAM5 promotes mitochondrial quality control through biogenesis and selective mitophagy under mild damage, but excessive activation under severe injury leads to mitochondrial dysfunction and cell death [68]. Our findings revealed that PGAM5 overexpression contributes to DON-induced mitochondrial dysfunction and excessive mitophagy. Recent evidence has indicated that PGAM5 modulates ferroptosis mainly by influencing mitochondrial dynamics and redox balance [44]. Specifically, PGAM5 overexpression enhances mitochondrial fragmentation, disrupts iron homeostasis, and promotes ROS accumulation, thereby facilitating lipid peroxidation and ferroptosis [69]. Our results revealed that LYC alleviated the DON-induced up-regulation of PGAM5 protein expression. However, PGAM5 overexpression abolished the protective effects of LYC, highlighting PGAM5 as a crucial mediator for restoring redox equilibrium and mitochondrial integrity. PGAM5 deletion is associated with altered mitochondrial dynamics characterized by increased fusion and decreased turnover, ultimately contributing to the premature senescence of retinal pigment epithelial cells [28]. Previous studies have shown that PGAM5 is linked to cardiac ischemia–reperfusion injury through mitochondrial quality control [67]. Recent reports have linked abnormal PGAM5 regulation to the development of intestinal diseases [70]. In summary, our findings suggested that LYC mitigated DON-induced intestinal barrier dysfunction by inhibiting PGAM5-mediated mitophagy-dependent ferroptosis, thereby preserving intestinal epithelial homeostasis.

Mitochondrial dysfunction and ferroptosis are increasingly recognized as key pathological features of inflammatory bowel disease. Our findings identify PGAM5-mediated mitophagy-dependent ferroptosis as a critical event in DON-induced intestinal epithelial injury. Theoretically, given the close association between DON and intestinal inflammation, this mechanism may help us understand the epithelial dysfunction in inflammatory bowel diseases. Although the vitro model is well suited for mechanistic investigations of the intestinal epithelial barrier, they still struggle to fully replicate the complex intestinal microenvironment in vivo. Therefore, animal models and clinical studies are needed to further validate the physiological importance of the PGAM5 signaling axis in pathological conditions, such as inflammatory bowel diseases. We also acknowledge several other limitations of the present study. First, the gut microbiota plays a crucial role in maintaining the stability of intestinal epithelium, and is closely related to the metabolism of mycotoxins and intestinal inflammation [71,72]. This study did not explore the impact of DON on the intestinal microbiota and its interaction with the epithelial response. This is an important area for future research. Second, with respect to the transfection experiments using mRFP-GFP-LC3 and mito-Keima, the inclusion of customized empty viral vector controls in future studies would help to exclude potential vector-associated effects. In addition, the temporal and causal relationships between mitophagy and ferroptosis have not been fully addressed. Our study has confirmed that PGAM5-mediated excessive mitophagy promotes the occurrence of ferroptosis. However, there is a feedback regulation between the two, and time-course analyses or stage-specific inhibitors are needed to further elucidate these interactions. Finally, the relatively low bioavailability of LYC as a lipophilic compound remains a limitation, and strategies to improve its delivery efficiency warrant further investigation.

## Conclusion

In conclusion, these findings demonstrated that, in the IPEC-J2 cell model, DON results in abnormal PGAM5 expression, which triggers excessive mitophagy and ferroptosis, ultimately leading to intestinal barrier dysfunction. However, LYC treatment successfully counteracted these alterations. From a mechanistic standpoint, LYC inhibits PGAM5-mediated mitophagy-dependent ferroptosis, thereby alleviating DON-induced intestinal barrier injury. Importantly, we confirmed that PGAM5 overexpression abolished the protective effects of LYC and restored the DON-induced elevation of mitophagy and ferroptosis, indicating that PGAM5 is a key molecule through which LYC exerts its antagonistic effects against DON-induced intestinal injury. Given the pivotal role of PGAM5, our findings reveal a novel mechanism by which PGAM5 may serve as a potential therapeutic target for the prevention and treatment of intestinal diseases.

## Materials and Methods

### Cell culture and treatment

IPEC-J2 cells were acquired from the laboratory of Prof Dong Na (Northeast Agricultural University, Harbin, China) and were cultured in Dulbecco’s modified Eagle’s medium (DMEM)/F12 (1:1) (MeilunBio, China) containing 10% fetal bovine serum (Cellmax, China) and 1% penicillin–streptomycin (Seven, Beijing, China). The IPEC-J2 cells were maintained at 37 °C in an atmosphere containing 5% CO_2_. LYC (Sigma-Aldrich, USA) was dissolved in tetrahydrofuran containing 0.025% hydroxytoluene at a concentration of 1 mM as a stock solution. The final concentration of tetrahydrofuran in the culture medium did not exceed 0.1% (volume/volume). DON (NCS Testing Technology Co., Ltd., Beijing, China) was solvated in DMSO to obtain a 4 mM stock solution, with the final DMSO concentration maintained below 0.1%. Based on previous studies and CCK-8 assays, LYC and DON were applied at concentrations of 1 μM for 24 h in subsequent experiments [65,73]. The IPEC-J2 cells were divided into 4 groups: CON (control), LYC (1 μM LYC), DON (1 μM DON), and LDO (1 μM LYC + 1 μM DON). For cellular stimulation, IPEC-J2 cells were treated with different reagents, including 10 μM Erastin, 10 μg/ml Pep-A, 10 μg/ml E64d, 20 nM Baf-A1, 100 nM RaPa, 2 μM Fer-1, and 5 mM 3-MA (MCE, New Jersey, USA). These conditions were selected based on preliminary cytotoxicity assessments and published literature to ensure effective pathway modulation without inducing nonspecific cell death [74,75].

### Ultrastructural observation of IPEC-J2 cells

The harvested cells were preserved in 2.5% glutaraldehyde, and followed by dehydration, soaking, embedding, ultrathin sectioning, lead citrate staining, and washing [76,77]. Furthermore, transmission electron microscopy (HT7650, Hitachi, Japan) was employed to capture images. The volume density of mitochondria was quantified, and mitochondrial structural damage was evaluated using the Flameng scoring system based on a previously study [78]. Detailed steps are provided in the Supplementary Methods 1.1. and 1.2.

### RNA-seq

Total RNA was extracted from cells following standard protocols, and subsequently constructed mRNA libraries, sequence, and analysis data [73,79]. The sequencing work was executed by Shanghai Paisennuo Biotechnology Co. Ltd. (Shanghai, China). Detailed descriptions of methodologies are listed in Supplementary Methods 1.3.

### Cell transfection

The PGAM5 overexpression plasmid (pcDNA3.1-PGAM5) were synthesized by Saiwen Innovation Biological Technology Co., Ltd. (Beijing, China). In brief, pcDNA3.1-PGAM5 and Lipofectamine 3000 (Invitrogen, USA) were individually diluted in Opti-MEM and subsequently mixed to generate transfection complexes. These complexes were applied to cells to achieve PGAM5 overexpression. In addition, an empty plasmid vector control (pcDNA3.1) was transfected and served as the negative control.

The siRNA targeting PGAM5 (sequence: GGCUGUGUAUUACGAAGAU) was synthesized by Saiwen Innovation Biological Technology Co., Ltd. (Beijing, China). The siPGAM5 and Lipofectamine 3000 (Invitrogen, USA) were separately diluted in Opti-MEM medium and then mixed to form transfection complexes, followed by introduced into IPEC-J2 cells. In addition, siNC stands for siRNA negative control.

### Cell viability assay

Cell viability was evaluated using the CCK-8 assay as previously described [80]. Briefly, cells were seeded into 96-well plates and allowed to adhere. After the indicated treatments, CCK-8 reagent was added to each well and the plates were incubated at 37 °C for 1 h. The absorbance at 450 nm was subsequently measured using a microplate reader (BioTek, Shanghai, China) to determine cell viability.

### GSH content assay

T-GSH levels were measured using a T-GSH microplate assay kit (Nanjing Jiancheng Bioengineering Institute, China) according to the manufacturer’s instructions. Briefly, cells were harvested after the indicated treatments and washed with phosphate-buffered saline (PBS). The supernatants were collected for T-GSH determination. The absorbance was recorded at 405 nm using a microplate reader (BioTek, Shanghai, China).

### ROS determination

A ROS assay kit (Beijing Boxbio Science & Technology Co., Ltd., Beijing, China) was used to measure the ROS concentration. Briefly, the cells were treated with DCFH-DA for the appropriate time. After incubation, the detection of DCF fluorescence was subsequently carried out using a flow cytometer (Solaibao, China).

### Assessment of MMP

According to the manufacturer’s directions, JC-1 fluorescent probe (Beyotime, Shanghai, China) was applied to evaluate MMP. The fluorescence was captured with a fluorescent microscope (Leica, Germany).

### Determination of TEER

The TEER was determined with the Millicell resistance system (Billerica, MA) to evaluate the barrier integrity of IPEC-J2 cells. Briefly, the cell suspension was added to the upper section of the transwell inserts (Jet Biofil, China), with the lower chamber containing DMEM/F12 medium. TEER values were recorded once daily during monolayer formation. The establishment of a confluent and functional epithelial monolayer was confirmed to be complete when resistance values reached a stable baseline plateau [81]. After the cell monolayers formed, cell treatments were applied, and the TEER values were recorded at 0, 12, 24, 36, and 48 h. The TEER was calculated according to the following Eq. (1):

$$\begin{array}{c} \text{TEER}\;\left(\Omega \cdot {\mathrm{cm}}^{2}\right)=\left(\text{treatment TEER}-\text{blank TEER}\right)\\ \;\times \text{membrane area}\;\left({\mathrm{cm}}^{2}\right) \end{array}$$ (1)

### Detection of cell permeability

The paracellular flux of FD-4 (Sigma-Aldrich, USA) in cells was measured as an indicator of cell permeability. FD-4 solution was added to the apical chamber of a transwell after 1 h of incubation, and the basolateral medium was transferred to 96-well plates. The fluorescence intensity was subsequently assessed using a multifunctional microplate reader (Shanghai, China) with excitation and emission wavelengths of 493 and 533 nm, respectively. FD-4 permeability was calculated using a normalization method, in which the fluorescence values were normalized to those of the corresponding control group, and the results are expressed as relative FD-4 permeability.

### Cell mobility analysis

The evaluation of cell migration was conducted using the scratch wound healing assay. A linear scratch was applied to the confluent cells, which were respective treatments and then wound closure was monitored using an EVOS imaging system (Invitrogen, USA) and quantification utilized the ImageJ software.

### Cytoskeleton analysis

The cytoskeletal components (F-actin and α-tubulin) were visualized using iFluor 488 phalloidin (Yeasen, China) and tubulin-tracker red (Beyotime, China). After fixation, the cells were stained with both probes. Fluorescence images were subsequently captured with a Leica fluorescence microscope (Germany).

### Detection of MitoSOX levels

The detection of mitochondrial ROS was performed with MitoSOX Red (Invitrogen, USA). IPEC-J2 cells were incubated with medium supplemented with MitoSOX Red solution for 10 min. The cells were examined and photographed using a fluorescence microscope (Leica, Germany).

### Assessment of lipid peroxidation

The assessment of lipid peroxidation levels in intracellular was applied with BODIPY fluorescent probe (Invitrogen, USA). In brief, cells were cultured with BODIPY working solution maintained in the dark for 30 min, followed by PBS washing. The fluorescence signal was imaged with a fluorescence microscope (Leica, Germany).

### Ferrous ion concentration determination

FerroOrange and Mito-FerroGreen (Dojindo Molecular Technologies, Japan) were employed to assess the concentration of cellular and mitochondrial ferrous iron. In short, the cells were treated as indicated and stained with FerroOrange and Mito-FerroGreen, respectively. Images were captured using a fluorescence microscope (Leica, Germany).

### mRFP-GFP-LC3 adenovirus transfection

Autophagy was detected using mRFP-GFP-LC3 adenovirus transfection (Hanbio Technology Co., Ltd., Shanghai, China). The cells were infected with adenovirus to visualize autophagosome formation, and fluorescence signals were visualized with a confocal microscope (Leica, Germany).

### Mito-Keima detection

Mitophagy rates were evaluated using mito-Keima (Hanbio Technology Co., Ltd., Shanghai, China) via adenoviral transfection, enabling the visualization and quantification of mitophagy activity. Fluorescence signals were recorded with a confocal microscope (Leica, Germany), and mitophagy was quantified.

### Western blot analysis

The extraction of total protein from cells was performed using radio immunoprecipitation assay lysis buffer (MeilunBio, China) containing phenylmethylsulfonyl fluoride (Seven, Beijing, China) and protease inhibitor cocktail (MedChem Express, USA), as described previously [82–84]. Proteins in equal quantities were separated using sodium dodecyl sulfate–polyacrylamide gel electrophoresis (Yeason, China) and then transferred onto nitrocellulose membranes. After being sealed with 5% nonfat milk for 2 h, the membranes were exposed to primary antibody (Affinity, USA; GeneTex, USA; Proteintech, USA; ABclonal Technology, China; Bioss, China) overnight at 4 °C, followed by the incubation of secondary antibodies (Zhongshan Jinqiao, China). The visualization of the protein bands was carried with an Amersham Imager (GE, Switzerland) and ImageJ software was employed to quantify the protein band density.

### IF analysis

IF staining was carried out following the methods outlined in prior research [85], with minor modifications. In brief, the cells underwent fixation with 4% paraformaldehyde, permeabilization with 0.5% Triton X-100, and blocking with 5% bovine serum albumin. Subsequently, the cells were treated with primary antibodies at 4 °C overnight, and then labeled with fluorescent secondary antibodies. The fluorescence images were acquired using a microscope (Leica, Germany).

### Molecular docking and dynamics analyses

Molecular docking was carried out with the Autodock Vina program, while GROMACS 2020 software was used for molecular dynamics simulation. The PyMOL program was employed to visually examine the docking outcomes. Detailed procedures are indicated in Supplementary Methods 1.4.

### Cellular thermal shift assay

The CETSA experiments were performed as previously described [86]. Briefly, the cells were lysed in PBS supplemented with protease inhibitors by ultrasonication. The protein stability of the resulting supernatants was determined using Western blot analysis.

### Statistical analysis

GraphPad Prism 9 software was used to analyze the experimental data. Schematic diagrams were created using BioRender (BioRender, Toronto, Canada). The professional language editing of this manuscript was provided by AJE (American Journal Experts, USA). All experiments were performed at least 3 times, and the results are presented as mean ± SD. Statistical analyses were performed using Student *t* test for comparisons between 2 groups, while comparisons among multiple groups were conducted using one-way analysis of variance, followed by Tukey’s post hoc pairwise comparison. Statistical significance was defined as *P* < 0.05.

## Data Availability

The data that support the findings of this study are available from the corresponding author upon reasonable request.
