# Supplementary material for: Lycopene Alleviates Deoxynivalenol-Induced Porcine Intestinal Epithelial Barrier Injury by Inhibiting PGAM5-Mediated Mitophagy-Dependent Ferroptosis
Source: Research (Wash D C). 2026 Apr 22;9:1251. doi: 10.34133/research.1251 (PMC13100353; doi:10.34133/research.1251)
Supplement: Supplementary 1 — Figs. S1 to S3 [file research.1251.f1.docx]

**Supporting Information**

**Title**

Lycopene alleviates deoxynivalenol-induced porcine intestinal epithelial barrier injury by inhibiting PGAM5-mediated mitophagy-dependent ferroptosis

**Authors**

Jing Zheng^a, 1^, Zi-Yan Hu^a, 1^, Ming Lou^a, 1^, Yue Cheng^a^, Yi-Feng Huang^a^, Ming-Shan Chen^a^, Jia-Xin Wang^a^, Fu-Wei Jiang^a^, Yi Zhang^a^, Zhuo-Yu Liu^a^, Si-Tong Liu^a^, Hong-Li Si^a^, Qi Yu^a^, Xiao-Yi Zhang^a^, Jin-Long Li^a, b, c^, Yi Zhao^a, b, c, *^

**Affiliations**

^a^ College of Veterinary Medicine, Northeast Agricultural University, Harbin 150030, P.R. China

^b^ Key Laboratory of the Provincial Education Department of Heilongjiang for Common Animal Disease Prevention and Treatment, Northeast Agricultural University, Harbin 150030, P.R. China

^c^ Heilongjiang Key Laboratory for Laboratory Animals and Comparative Medicine, Northeast Agricultural University, Harbin 150030, P.R. China

^*^Corresponding author.

Yi Zhao

Address: College of Veterinary Medicine, Northeast Agricultural University, Harbin, 150030, P. R. China

Tel: +86 451 55190407

E-mail address: Zhaoyi@neau.edu.cn (Y. Zhao)

^1^ These authors contributed equally to this study

1. **Supporting Figures**

**
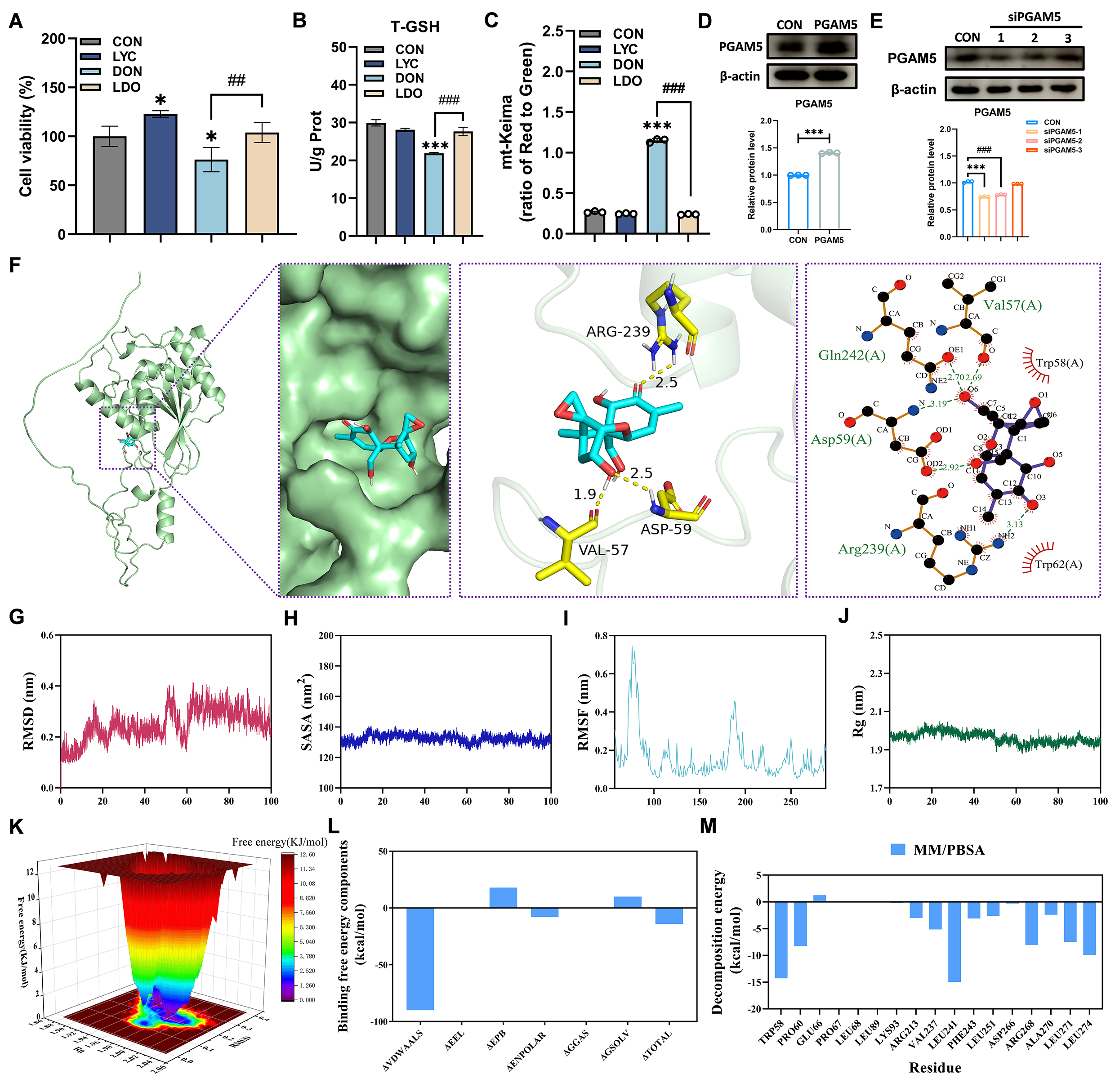
**

**Fig. S1.** PGAM5 knockdown alleviated DON-induced impairment of intestinal barrier function by inhibiting ferroptosis and autophagy. (A) The CCK8 assay. (B) T-GSH content. (C) The ratio of Red to Green fluorescence of mt-Keima. (D) Western blot analysis and quantitative results of PGAM5 protein expression in CON and PGAM5 overexpression groups. (E) Western blot analysis and quantitative results of PGAM5 protein expression in CON and si-PGAM5 transfection groups (siPGAM5-1/2/3: three different siRNA sequences targeting PGAM5). (F) Molecular docking simulation for the ligand–protein binding of DON with PGAM5. (G) RMSD of the complex. (H) SASA. (I) RMSF number. (J) Rg number. (K) The Gibbs energy landscape of complex. (L) Binding free energy components. (M) Decomposition energy. Data are presented as the mean ± SD, n=3. Symbol for the significance of differences between the CON group and LYC group, DON group, PGAM5 group or siPGAM5-1 group: *^*^P <* 0.05*, ^***^P <* 0.001. Symbol for the significance of differences between the DON group and LDO group, or the CON group and siPGAM5-2 group: *^##^P < 0.01, ^###^P <* 0.001.

**
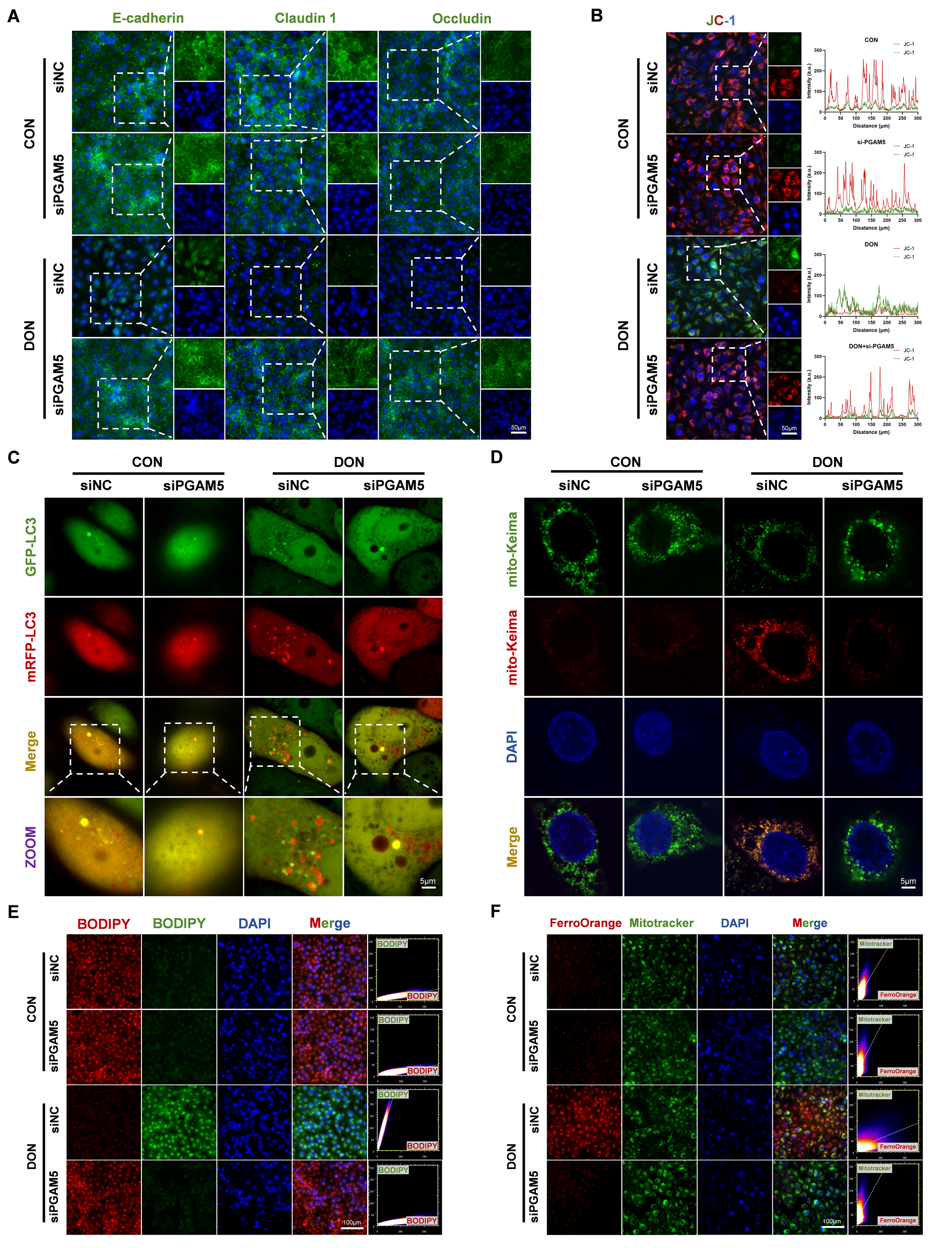
**

**Fig. S2.** PGAM5 knockdown alleviated DON-induced impairment of intestinal barrier function by inhibiting ferroptosis and autophagy. (A) Representative IF images of TJ-related proteins. (B) Representative IF images of JC-1. (C) mRFP-GFP-LC3 adenovirus transfection. (D) mito-Keima adenovirus transfection. (E) Lipid ROS level. (F) Intracellular iron level.

**
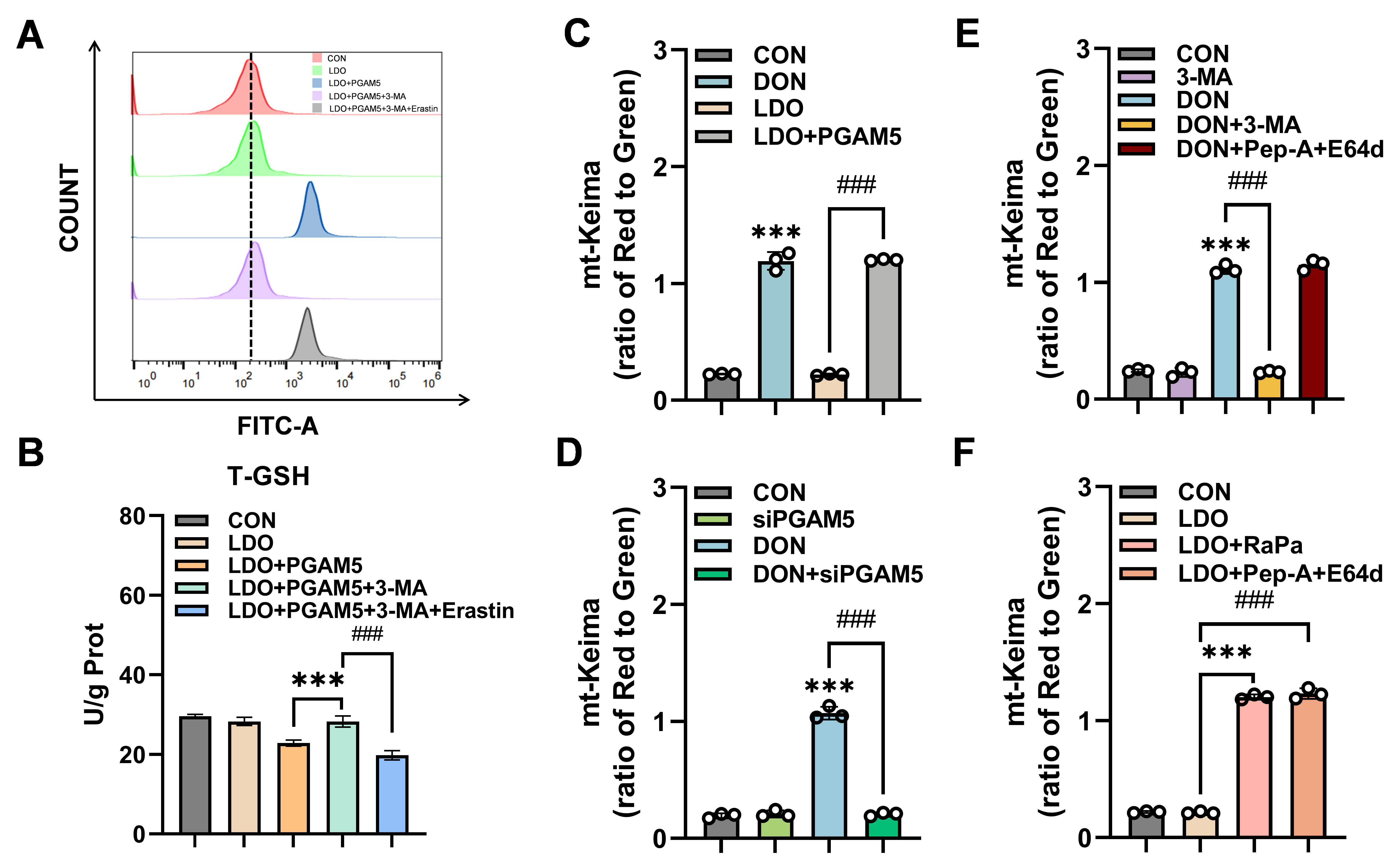
**

**Fig. S3.** PGAM5 overexpression eliminated the antagonistic effects of LYC on DON-induced ferroptosis. (A) ROS level. (B) T-GSH content. (C, D, E and F) The ratio of Red to Green fluorescence of mt-Keima. Data are presented as the mean ± SD, n=3. Symbol for the significance of differences between the LDO+PGAM5 group and LDO+PGAM5+3-MA group, the CON group and DON group or the LDO group and LDO+RaPa group: *^***^P <* 0.001. Symbol for the significance of differences between the LDO+PGAM5+3-MA group and LDO+PGAM5+3-MA+Erastin group, the LDO group and LDO+PGAM5 group, the DON group and DON+siPGAM5 group, the DON group and DON+3-MA group, or the LDO group and LDO+Pep-A+E64d group: *^###^P <* 0.001.

**Supplementary 1. Details of Materials and Methods**

**Supplementary Methods 1.1. Mitochondrial volume density**

A method has used to quantify mitochondria consists of a method known as “point counting grids”. Simply put, this is done by placing a transparent square lattice grid over the electron micrograph. The grids normally have lines that intersect one another at a 90° angle and are separated from each other by 1 cm. A lattice of 100 points is normally useful for micrograph. The number of times a mitochondrion located at a line intersection was recorded. By knowing the total amount of intersects that are possible with the lattice grid and the number of times a mitochondrion intersected a line intersect, this number is divided by the total.

**Supplementary Methods 1.2 Flameng score**

Flameng score is a scoring standard used to analyze the degree of mitochondrial damage. The increase of the Flameng score showed the mitochondrial damage. The mitochondrial Flameng score criteria under transmission electron microscope are as follows. The greater the IPEC-J2 cells mitochondria damage, the higher the score and injury is scored as 0-4 points: 0, the structure of mitochondria is normal and they are full of particles; 1, the structure of mitochondria is essentially normal, but the matrix particles are lost; 2, mitochondrial swelling and matrix transparency are apparent; 3, rupture of mitochondrial cristae with matrix transparency and concentration; 4, the mitochondrial cristae are split, the integrity of the mitochondria inside and outside the membrane has been lost, and they appear vacuolated.

**Supplementary Methods 1.3 RNA-seq analysis**

Total RNA was isolated using a TRIzol total RNA extraction kit (TIANGEN, Cat. No. DP424), which yielded > 2 μg of total RNA per sample. RNA quality was examined by 0.8% agarose gel electrophoresis and spectrophotometry. High-quality RNA with a 260/280 absorbance ratio of 1.8-2.2 was used for library construction and sequencing. Illumina HiSeq library construction was performed according to the manufacturer's instructions (Illumina,USA). Oligo-dT primers are used to reverse transcribe mRNA to obtain cDNA (APExBIO, Cat. No. K1159). Amplify cDNA for the synthesis of the second chain of cDNA. Purify cDNA products by magnetic beads. After library construction, library fragments were enriched by PCR amplification and selected according to a fragment size of 350-550 bp. The library was quality-assessed using an Agilent 2100 Bioanalyzer (Agilent, USA). The library was sequenced using the Illumina NovaSeq 6000 sequencing platform (Paired end150) to generate raw reads. Raw paired-end fastq reads were filtered by TrimGalore to discard the adapters and low-quality bases via calling the Cutadapt tool. The clean reads obtained were then aligned to the pig reference genome using HISAT2, followed by reference genome-guided transcriptome assembly and gene expression quantification using StringTie. Differentially expressed genes (DEGs) were identified by DEseq2 (for sample with replications) or edgeR (for sample with no replication) with a cut-off value of log2|fold-change| > 1 and p-adjust < 0.05. The clusterProfiler was used to perform functional enrichment analysis for the annotated KEGG pathway categories. Terms with *p < 0.05* were considered significant.

**Supplementary Methods 1.4 Molecular docking and Molecular Dynamics Simulations**

PubChem and PDB were used to find the chemical and conformational information of the relevant proteins (PGAM5) and small-molecule compounds (DON/LYC). The AutoTools software was used to remove the redundant protein chains, ligands and water molecules with hydrogenation before running docking experiments. The AutoGrid software was used to calculate the energy lattice points. AutoDock Vina was used to simulate the docking condition between proteins (PGAM5) and small molecules (DON/LYC). Autogrid calculations were performed, selecting AutoDock Vina as the docking algorithm, and AutoDock was used for molecular docking. The binding energy results were visualized using PyMOL.

The resulting proteins from docking were separated from the best small molecule ligands, and small molecule force field files were generated by the antechamber tool in AmberTools software, and the small molecule force field files were converted into gromacs force field files by the acpype software tool. GAFF force field was used for small molecules, while AMBER14SB force field and TIP3P water model were used, and the files of protein and small molecule ligand were combined to construct the simulation system of the complex. Molecular dynamics simulations (MD) were performed using GROMACS 2022 program under constant temperature and periodic boundary conditions. During the MD simulation, all involved hydrogen bonds were constrained using the LINCS algorithm with an integration step size of 2 fs. The electrostatic interactions were calculated by the (Particle-mesh Ewald) PME method and the cutoff value was set to 1.2 nm. The non-bonded interaction cutoff was set to 10 Å and updated every 10 steps. The simulation temperature was controlled at 298 K using the V-rescale temperature coupling method and the Berendsen method to control the pressure at 1 bar. At 298 K, 100 ps, NVT, equilibration simulation with NPT, and 100 ns, MD simulation of the complex system, preserving the conformation every 10 ps. After the simulation, the simulated trajectories were analyzed using VMD and Pymol and MMPBSA binding free energy analysis between the protein and small molecule ligands using the g_mmpbsa program.
